# Seedless growth of zinc oxide flower-shaped structures on multilayer graphene by electrochemical deposition

**DOI:** 10.1186/1556-276X-9-337

**Published:** 2014-07-05

**Authors:** Nur Suhaili Abd Aziz, Tomoya Nishiyama, Nurul Izni Rusli, Mohamad Rusop Mahmood, Kanji Yasui, Abdul Manaf Hashim

**Affiliations:** 1 Malaysia-Japan International Institute of Technology, Universiti Teknologi Malaysia, Jalan Semarak, Kuala Lumpur 54100, Malaysia; 2 Department of Electrical Engineering, Nagaoka University of Technology, Kamitomioka-machi, Nagaoka, Niigata 940-2137, Japan; 3 Faculty of Engineering Technology, Universiti Malaysia Perlis, Uniciti Campus Sungai Chucuh, Padang Besar, Perlis 02100, Malaysia; 4 Faculty of Electrical Engineering, Universiti Teknologi MARA, Shah Alam, Selangor 40540, Malaysia; 5 MIMOS Berhad, Technology Park Malaysia, Kuala Lumpur 47000, Malaysia

**Keywords:** Graphene, Zinc oxide, Electrochemical deposition, Flower-shaped structure, Rod

## Abstract

A seedless growth of zinc oxide (ZnO) structures on multilayer (ML) graphene by electrochemical deposition without any pre-deposited ZnO seed layer or metal catalyst was studied. A high density of a mixture of vertically aligned/non-aligned ZnO rods and flower-shaped structures was obtained. ML graphene seems to generate the formation of flower-shaped structures due to the stacking boundaries. The nucleation of ZnO seems to be promoted at the stacking edges of ML graphene with the increase of applied current density, resulting in the formation of flower-shaped structures. The diameters of the rods/flower-shaped structures also increase with the applied current density. ZnO rods/flower-shaped structures with high aspect ratio over 5.0 and good crystallinity were obtained at the applied current densities of −0.5 and −1.0 mA/cm^2^. The growth mechanism was proposed. The growth involves the formation of ZnO nucleation below 80°C and the enhancement of the growth of vertically non-aligned rods and flower-shaped structures at 80°C. Such ZnO/graphene hybrid structure provides several potential applications in sensing devices.

## Background

In recent years, the concept of advanced heterogeneous integration on silicon (Si) platform has attracted much attention towards the realization of a ‘More than Moore’ technology [1]. To realize such technology, the growth of high-quality elements (i.e., germanium (Ge) [2]) compound semiconductors (i.e., gallium arsenide (GaAs) [3], gallium nitride (GaN) [4], silicon carbide (SiC) [5]), metal oxides (i.e., zinc oxide (ZnO) [6]), and carbon-based materials (i.e., graphene [7], carbon nanotube (CNT) [8]) on Si platform is highly required. The co-integration of these materials enables the present ultra-large-scale integrated circuits (ULSIs) to be facilitated not only with ultra-high speed complementary metal-oxide semiconductor (CMOS) transistors and novel transistors [9] but also with various kinds of functional devices, such as optical devices [10], photodetectors [11], solar batteries [12], and sensors [13,14]. Such intelligent system-on-chip (i-SoC) on Si is considered as a promising and practical direction.

ZnO is a promising candidate for the fabrication of several kinds of devices due to its unique properties such as wide bandgap and large exciton energy. In order to fabricate ZnO-based devices on Si substrate, it is necessary to electronically isolate both materials using an insulator such as silicon dioxide (SiO_2_). Therefore, a breakthrough on the growth technology is strongly required to realize a high-quality ZnO-on-insulator structure with excellent crystallinity since the insulator is amorphous and the lattice mismatch is relatively large. There are several reports on the growth of ZnO nanostructures on insulators such as SiO_2_[15,16], but the densities of the grown ZnO nanostructures were very low. Therefore, the ZnO seed layer is commonly used as the nucleation site to enable the subsequent growth of ZnO nanostructures on insulators [17-20].

Graphene is a two-dimensional hexagonal network of carbon atoms which is formed by making strong triangular σ-bonds of the sp^2^ hybridized orbitals. Since the bonding structure of graphene is similar to the *C* plane of the hexagonal crystalline structure of ZnO, it seems to be feasible for graphene to serve as an excellent template layer for the growth of high-density ZnO nanostructures on the insulator. In addition, since graphene is an excellent conductor and transparent material, the hybrid structure of a ZnO nanostructure and graphene shall lead to several device applications not only on Si substrate but also on other insulating substrates such as glass and flexible plastic. For examples, such hybrid structure can be used for sensing devices [21], ultraviolet (UV) photodetectors [22], solar cells [23], hybrid electrodes for GaN light-emitting diodes (LEDs) [24], etc.

There are several potential methods to grow ZnO on graphene which can be categorized into vapor phase and liquid phase methods. Vapor phase method is likely to involve a high-temperature process and is also considered as a high-cost method [25]. Also, since the process requires oxygen (O_2_), the possibility of graphene to be oxidized or etched out during the growth is high since the oxidation of graphene is likely to occur at temperatures as low as 450°C [26,27]. Liquid phase method seems to be a promising method to grow graphene at low temperature with good controllability in terms of growth rates and structure dimensions.

To our knowledge, only two methods have been reported on the growth of seedless ZnO nanostructures on graphene via low-temperature liquid phase method. The term ‘seedless’ refers to the omission of pre-deposition of the ZnO seed layer by other processes and metal catalysts. Kim et al. reported the growth of ZnO nanorods on graphene without any seed layer by hydrothermal method, but the obtained results show low density of nanostructures [15]. Xu et al*.* reported the seedless growth of ZnO nanotubes and nanorods on graphene by electrochemical deposition [28,29]. They reported the growth of highly dense ZnO nanostructures by using solely zinc nitrate as the electrolyte with the introduction of oxidation process of graphene prior to actual growth. They also reported that the diameter, length, and morphology of the nanostructures showed significant dependencies on the growth parameters such as current density, precursor concentration, and growth time. Several other reports also indicated that current density plays an important role in inducing the growth of ZnO nanostructures on the seedless substrate [30,31]. Recently, Aziz et al. reported the electrodeposition of highly dense ZnO nanorods on single-layer (SL) graphene [30]. Furthermore, the distance between the electrodes and the molarity of electrolyte are also able to give significant effects on the properties of the resulting nanostructures [32]. Generally, a change in distance between the two electrodes can change the rate of the electrolysis reaction due to the change in the level of current density. The shorter the distance between the electrodes, the higher the electric field and thus the higher current density will be applied [32]. In this paper, we report the seedless growth of highly dense ZnO flower-shaped structures on multilayer (ML) graphene by a single-step cathodic electrochemical deposition method.

## Methods

**Figure 1:**
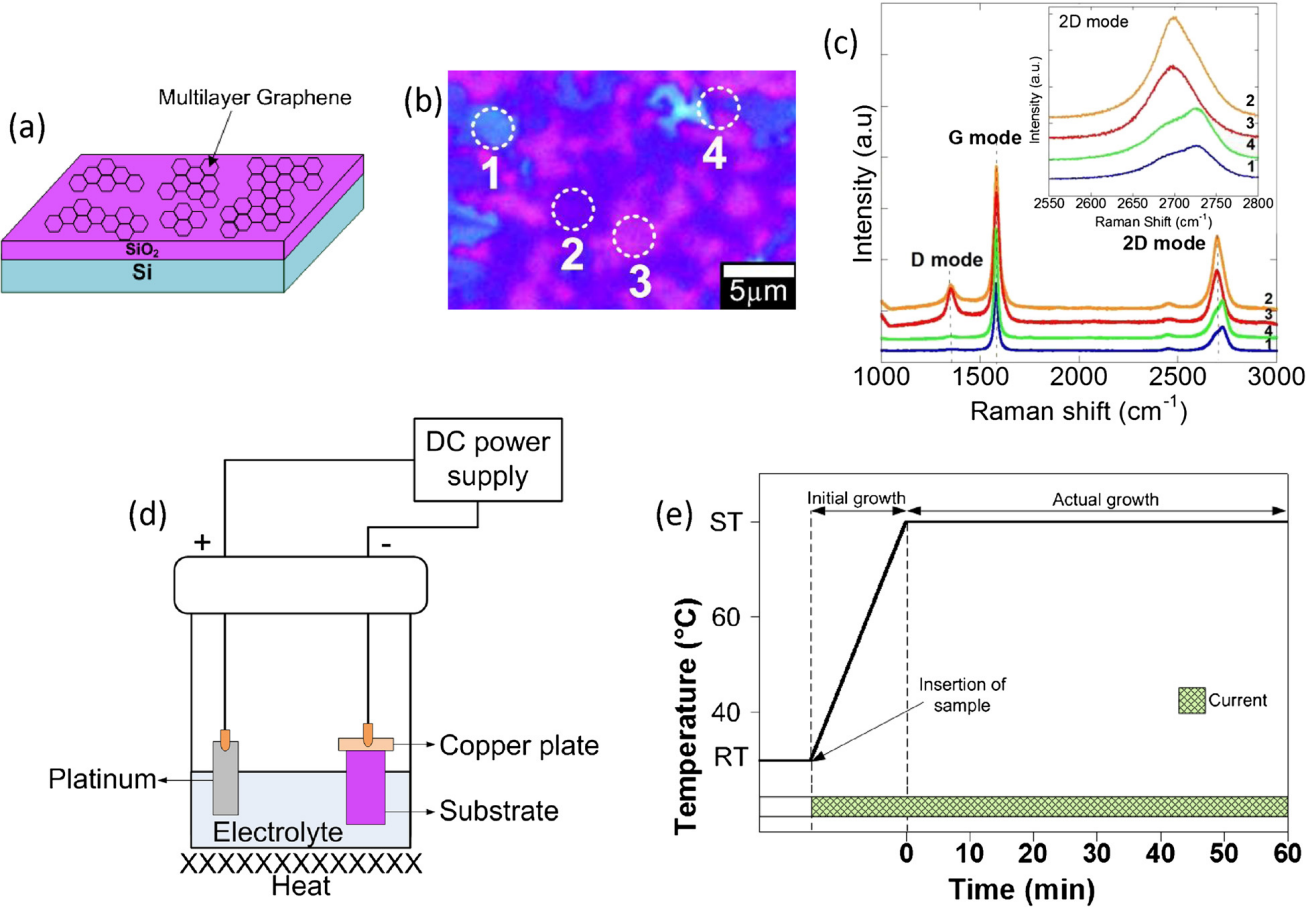
**CVD-grown ML graphene and electrochemical deposition. (a)** Schematic of ML graphene substrate, **(b)** Nomarski image of ML graphene, **(c)** Raman spectra for as-received ML graphene (the measured regions were identified in the circles), **(d)** schematic of electrochemical deposition setup, and **(e)** time chart for electrochemical growth process.

Figure 1a shows the schematic of chemical vapor deposition (CVD)-grown ML graphene on a SiO_2_/Si substrate (Graphene Laboratories Inc., Calverton, NY, USA). The Nomarski optical image of ML graphene in Figure 1b shows the visibility of graphene sheets on the SiO_2_/Si substrate with different numbers of layers [33] which is consistent with the measured Raman spectra shown in Figure 1c. Ferrari et al. reported that the two-dimensional (2D) peaks which occur at approximately 2,700 cm^−1^ for bulk graphite have much broader and upshifted 2D band which can be correlated to few-layer graphene [34].

The growth of ZnO structures on graphene/SiO_2_/Si was carried out by a cathodic electrochemical deposition in a mixture of 50 mM of zinc nitrate hexahydrate (Zn(NO_3_)_2_ · 6H_2_O; Sigma-Aldrich, St. Louis, MO, USA; ≥99.0% purity) and hexamethylenetetramine (HMTA, C_6_H_12_N_4_, Sigma-Aldrich, ≥99.0% purity). As shown in Figure 1d, platinum (Pt) wire acted as an anode (counter electrode) while graphene acted as a cathode. Both anode and cathode were connected to the external direct current (DC) power supply. In this experiment, the electrodeposition was operated under galvanostatic control where the current density was fixed during the deposition. It is noted here that the distance between the two electrodes was fixed at 4 cm for all experiments in order to avoid the other possible effects apart from the current density. The current densities of −0.1, −0.5, −1.0, −1.5, and −2.0 mA/cm^2^ were applied. All experiments were done by inserting the sample into the electrolyte from the beginning of the process or before the electrolyte was heated up from room temperature (RT) to 80°C. The actual growth was done for 1 h, counted when the electrolyte temperature reached 80°C or the set temperature (ST). Such temperature was chosen since the effective reaction of zinc nitrate and HMTA takes place at temperatures above 80°C. As reported by Kim et al., the activation energy to start the nucleation of ZnO cannot be achieved at temperatures below 50°C in such electrolyte [15]. After 1 h, the sample was removed immediately from the electrolyte and quickly rinsed with deionized (DI) water to remove any residue from the surface. The time chart of the growth is shown in Figure 1e.

The surface morphology, elemental composition, crystallinity, and optical properties of the grown ZnO structures were characterized using field emission scanning electron microscopy (FESEM), energy-dispersive X-ray spectroscopy (EDX), X-ray diffractometer (XRD), and photoluminescence (PL) spectroscopy with excitation at 325 nm of a He-Cd laser, respectively.

## Results and discussion

**Figure 2:**
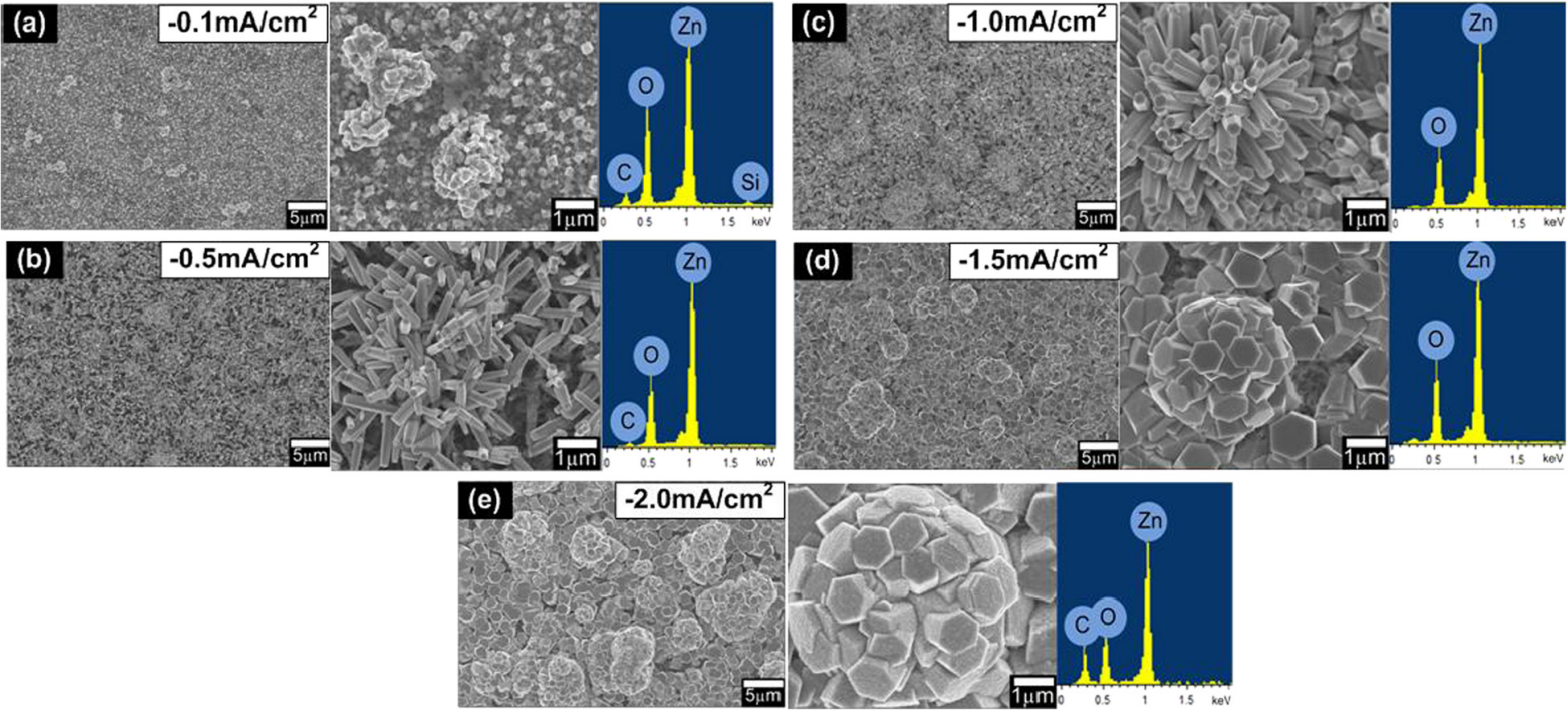
**Top-view and magnified images of FESEM and EDX spectra for ZnO structures.** The structures were grown at current densities of **(a)** −0.1 mA/cm^2^, **(b)** −0.5 mA/cm^2^, **(c)** −1.0 mA/cm^2^, **(d)** −1.5 mA/cm^2^, and **(e)** −2.0 mA/cm^2^.

Figure 2a,b,c,d,e shows the surface morphologies of the grown ZnO structures after 1 h of actual growth with their respective EDX spectra at current densities of −0.1, −0.5, −1.0, −1.5, and −2.0 mA/cm^2^, respectively. The ratio of Zn and O was found to show a value of more than 0.90 for all tested samples. This high ratio value seems to suggest that the synthesized ZnO structures have good stoichiometry.

It can be seen that the morphology of the grown ZnO at −0.1 mA/cm^2^ shows the formation of ZnO clusters. As the current density is changed from −0.5 to 2.0 mA/cm^2^, the morphology shows the mixture of vertically aligned/non-aligned ZnO rods and flower-shaped structures and their diameters or sizes increase with the current density. The formation of flower-shaped structures seems to be promoted by the stacking structures of ML graphene. This formation of flower-shaped structures was not observed for the growth of ZnO nanorods on oxidized bilayer graphene and SL graphene as reported by Xu et al. and Aziz et al., respectively [29,30]. The proposed growth mechanism is described in the next section.

The density of rods was determined by averaging the quantities of rods calculated at three different areas on each sample with a total area size of 125 μm^2^ for each area, and then, the obtained value was normalized to square centimeters (cm^2^). It is noted that the numbers of rods in such a large area size of 125 μm^2^ were obtained from the summation of rods contributed by five FESEM surface morphological images where each image had the area dimension of 5 μm × 5 μm. It is noted here that the actual density of each sample should be higher since the calculated quantity is not considering the unobservable rods of flower-shaped structures. Table 1 summarizes the density, diameter, length, and aspect ratio of the grown ZnO structures and the comparison with other works. Here, the calculated densities of rods for samples at current densities of −0.5, −1.0, −1.5, and −2.0 mA/cm^2^ were estimated to be around 7.95 × 10^8^, 7.11 × 10^8^, 1.67 × 10^8^, and 4.18 × 10^7^ cm^−2^, respectively. The density is 1 order larger than the density of nanorods grown by the hydrothermal method [15] and in the same order with the estimated nanorods grown by the electrochemical process on oxidized graphene layer reported by Xu et al*.* and on single-layer graphene reported by Aziz et al. [29,30]. The current applied in the electrochemical process seems to induce and promote the growth of ZnO rods/flower-shaped structures with high density.

**Table 1:** Density, diameter, length and aspect ratio of the grown ZnO rods

	**Current density (mA/cm**^**2**^**)**	**Density (cm**^**−2**^**)**	**Diameter of rods (nm)**	**Length of rods (nm)**	**Aspect ratio**
This work	−0.5	7.95 × 10^8^	170 to 240	810 to 1,220	5.10
	−1.0	7.11 × 10^8^	240 to 360	1,120 to 1,990	5.40
	−1.5	1.67 × 10^8^	900 to 1,160	400 to 840	0.55
	−2.0	4.18 × 10^7^	1,470 to 1,940	520 to 1,020	0.45
[15]	-	3.00 × 10^7^	680	1,400	2.10
[29]	−0.15	5.83 × 10^8^	370 to 780	-	-
	−0.1	1.84 × 10^7^	190 to 450	450 to 1,160	2.32
	−0.5	1.37 × 10^9^	260 to 480	840 to 1,160	2.70
[30]	−1.0	1.24 × 10^8^	660 to 1,000	150 to 340	0.28
	−1.5	3.42 × 10^7^	950 to 1,330	200 to 560	0.34
	−2.0	2.32 × 10^7^	570 to 2,030	1,160 to 2,220	1.14

**Figure 3:**
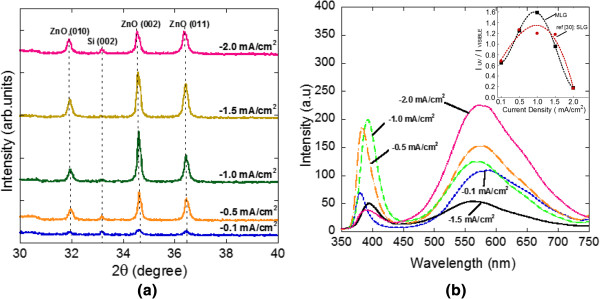
**XRD and PL spectra. (a)** XRD spectra and **(b)** RT PL spectra of grown ZnO structures at different applied current densities.

Figure 3a shows the XRD spectra of the as-grown ZnO rods on ML graphene at different current densities. The diffraction peaks of ZnO at approximately 31.94°, approximately 34.58°, and approximately 36.44° (reference code 98-008-1294, code 98-005-5014) were recorded which belong to (010), (002), and (011) planes, respectively. These diffraction peaks show that the grown ZnO nanostructures were having wurtzite structure [6]. Furthermore, there was also a weak peak at approximately 33.19° which corresponds to the Si (002) diffraction peak (reference code 98-007-9036). A relatively high peak intensity of the ZnO (002) plane observed in the samples grown at current densities of −0.5, −1.0, −1.5, and −2.0 mA/cm^2^ simply indicated the growth of vertically aligned ZnO rods along the *c*-axis. Meanwhile, the relatively high peaks corresponding to the ZnO (010) and (011) planes observed in those samples indicated the formation of vertically non-aligned rods and flower-shaped structures. These results are consistent with the SEM images shown in Figure 2. However, the observed weak peaks of the ZnO (002), (010), and (011) planes, particularly for the sample grown at a current density of −0.1 mA/cm^2^, justified the less formation of vertically aligned/non-aligned rods as well as flower-shaped structures.

Figure 3b shows the RT PL spectra of ZnO structures grown at different current densities. Here, two distinct emission bands were observed. The first band located in the UV region was estimated to be around 379, 385, 392, 395, and 389 nm for samples at current densities of −0.1, −0.5, −1.0, −1.5, and −2.0 mA/cm^2^, respectively. This band is claimed to be due to the near-band edge (NBE) emission or the recombinations of free excitons through an exciton-exciton collision process [6,29]. The second band appears in the green region of the visible spectrum at approximately 576, 574, 569, 563, and 569 nm, respectively. This band is commonly referred to as a deep-level or trap-state emission. Some researchers suggested that it could be attributed to the recombination of photogenerated holes with single ionized charge states of specific defects such as O vacancies or Zn interstitials [6,31,35]. However, Kang et al. reported that the singly ionized oxygen vacancy is responsible for the green emission and not the ionized Zn interstitials [36]. It is needed to be proved by post-annealing process of samples. Besides, the intensity of the peak also indicates the level of defects in the samples [31]. Surface state has also been identified as a possible cause of the visible emission in ZnO nanomaterials [37].

There are several reports discussing the relationship of these emission peaks with the quality of the grown structures. As been reported by Djurišić and Leung, the intensity of UV emission is dependent on the nanostructure size [38]. Below a certain size, the luminescence properties of the ZnO nanostructure should be dominated by the properties of the surface. The samples grown at current densities of −0.5 and −1.0 mA/cm^2^ show highly intense UV emission with the highest aspect ratio (Table 1) compared to other samples. Highly intense UV emission seems to show higher crystallinity and more perfection in surface states as reported by Park et al. [39]. Chen et al. suggested that it may imply a good crystal surface [40]. The enhancement of UV emission is attributed to a larger surface area and fewer defects [41]. Furthermore, the narrow peak with high intensity of NBE emission as well as a decrease in the peak density of green emission may indicate a high crystallinity of the grown structure [31,42]. It is also due to the vertical growth of ZnO rods and their high surface areas as suggested by Xu et al. [29]. The calculated ratio of the intensity of UV emission to the intensity of green emission, *I*_UV_/*I*_Visible_, obtained in this work is shown in Figure 3b (inset). As a comparison, the results obtained for the electrodeposition on SL graphene [30] were also plotted in the same figure. It can be seen that both spectra show a similar tendency. It can be seen that the sample grown on ML graphene at a current density of −1.0 mA/cm^2^ shows the highest value of 1.6 which seems to indicate the optimum current density for this work. The sample grown at a current density of −2.0 mA/cm^2^ shows the highest green emission compared to the other samples or the lowest *I*_UV_/*I*_Visible_ value, which indicates that there may be more defects induced during the growth such as O vacancies [43]. Ahn et al. reported that the sensitivity of gas sensing increases linearly with the sample having high green emission intensity or, in other words, with the structure having large defect density [14]. Therefore, it seems to suggest that the sample with large structural defect also has several interesting applications.

### Growth mechanism

**Figure 4:**
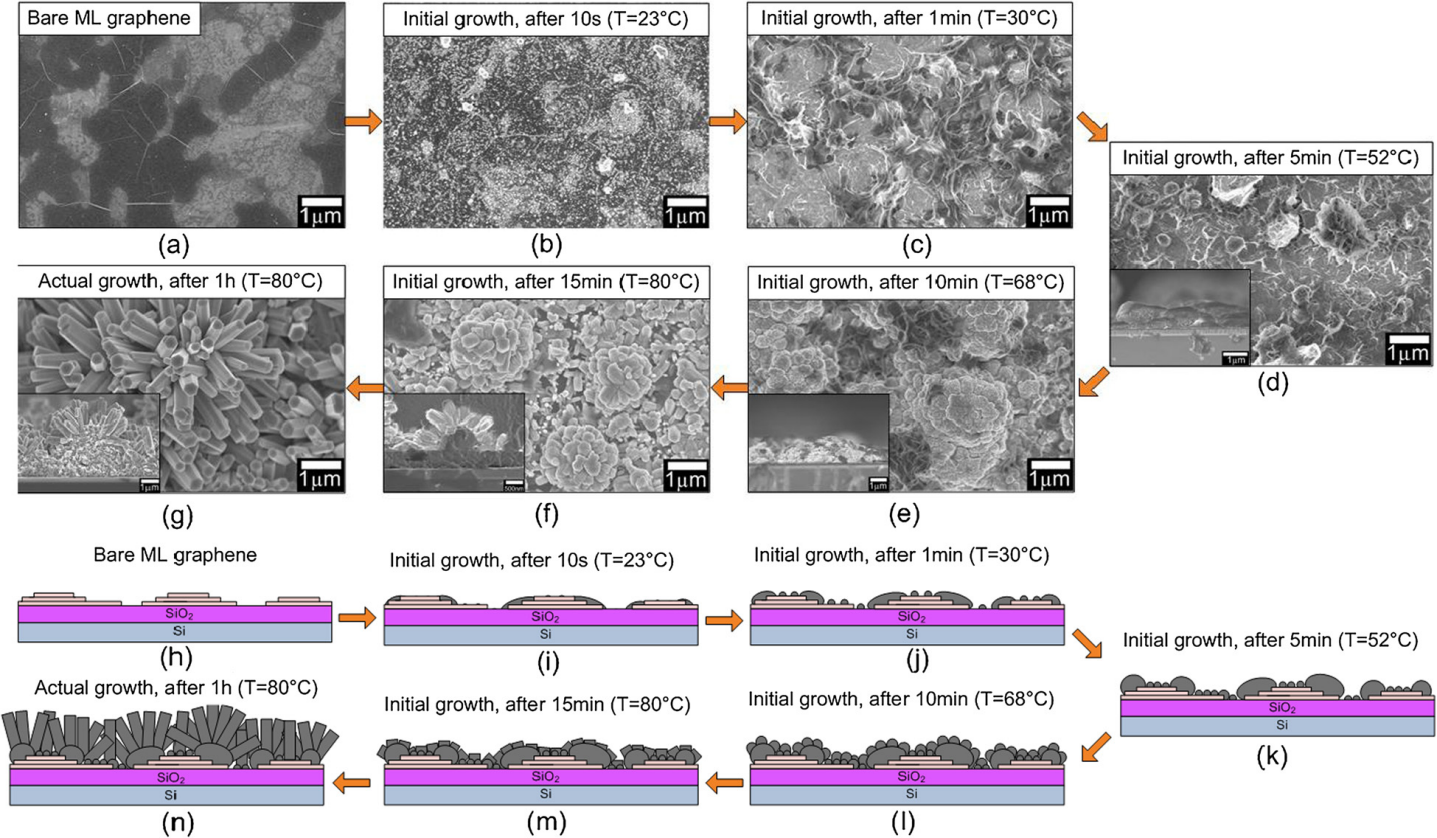
**FESEM images of bare ML graphene and ZnO structures grown on it at different growth times. (a)** Bare ML graphene. **(b, c, d, e, f)** ZnO structures grown on ML graphene after 10 s, 1 min, 5 min, 10 min, and 15 min of the initial growth, respectively. **(g)** ZnO structures grown on ML graphene after 1 h of the actual growth. **(h, i, j, k, l, m, n)** Schematics to illustrate the growth.

To understand the growth mechanism, we have investigated the surface and cross-sectional structures both at the initial stage of the growth, i.e., before reaching the ST point, and after 1 h of actual growth. As the procedure of a study at the initial growth, the samples were grown at several growth times, i.e., 10 s (*T* = 23°C), 1 min (*T* = 30°C), 5 min (*T* = 52°C), 10 min (*T* = 68°C), and 15 min (*T* = 80°C). The current was fixed at −1.0 mA/cm^2^. The current was immediately turned off after reaching these growth times, and at the same time, a sample was immediately taken out from the electrolyte and immersed into DI water to remove any residue. Figure 4a shows a FESEM image of bare ML graphene used in this work. It can be seen that the differences in contrast and brightness of the image represent the differences in thicknesses of graphene. The dark color shows the thicker graphene, while the bright color shows the thinner graphene. Figure 4b shows an image after the growth time of 10 s. It can be seen that the surface was covered with a high density of white ZnO cluster-like spots. This indicates that the nucleation of ZnO starts aggressively in a short time after the introduction of current even at a low temperature of 23°C. With the increase of growth time to 1 min (*T* = 30°C), it can be seen that almost the entire surface was covered with the ZnO thin layer with a rough morphology in different brightness levels, as shown in Figure 4c. It is noted here that the difference in brightness of the FESEM images represents the different thicknesses of the deposited ZnO structures. As the growth time and temperature were further increased to 5 min (*T* = 52°C), the nucleated ZnO structures become bigger and thicker and the entire surface was covered with ZnO, as shown in Figure 4d. However, there are also ZnO structures with small clusters formed at this stage. As shown in Figure 4e, the branching of ZnO rods on the large-sized ZnO clusters to form flower-shaped structures starts to take place when the growth time exceed 10 min (*T* = 68°C). On the other hand, the observation of vertically aligned/non-aligned individual rods may be generated from the ZnO structures with small cluster sizes. It can be seen in Figure 4f that the length of vertically aligned/non-aligned rods and flower-shaped structures increases with the growth time and temperature, but their diameters are showing no significant change. It can be concluded that the formation of flower-shaped structures has already taken place at the initial growth stage, i.e., before the ST point (below 80°C). Figure 4g shows the grown ZnO structures after 1 h of actual growth (at a constant temperature of 80°C). It clearly shows the increase in the lengths of rods, but the diameters are almost unchanged. The structures also show a well-defined hexagonal shape due to the effective decomposition of HMTA at 80°C to promote the formation of hexagonal ZnO structures. Figure 4h,i,j,k,l,m,n shows the schematics to illustrate the growth shown in Figure 4a,b,c,d,e,f,g, respectively. Since the reaction of electrolyte is considerably premature at temperatures below 80°C, the elemental composition of the seed structure is not good. This is proved by the EDX analysis for the samples grown after 15 min where the ratio of Zn and O is in the range of 0.5 to 0.6.

**Figure 5:**
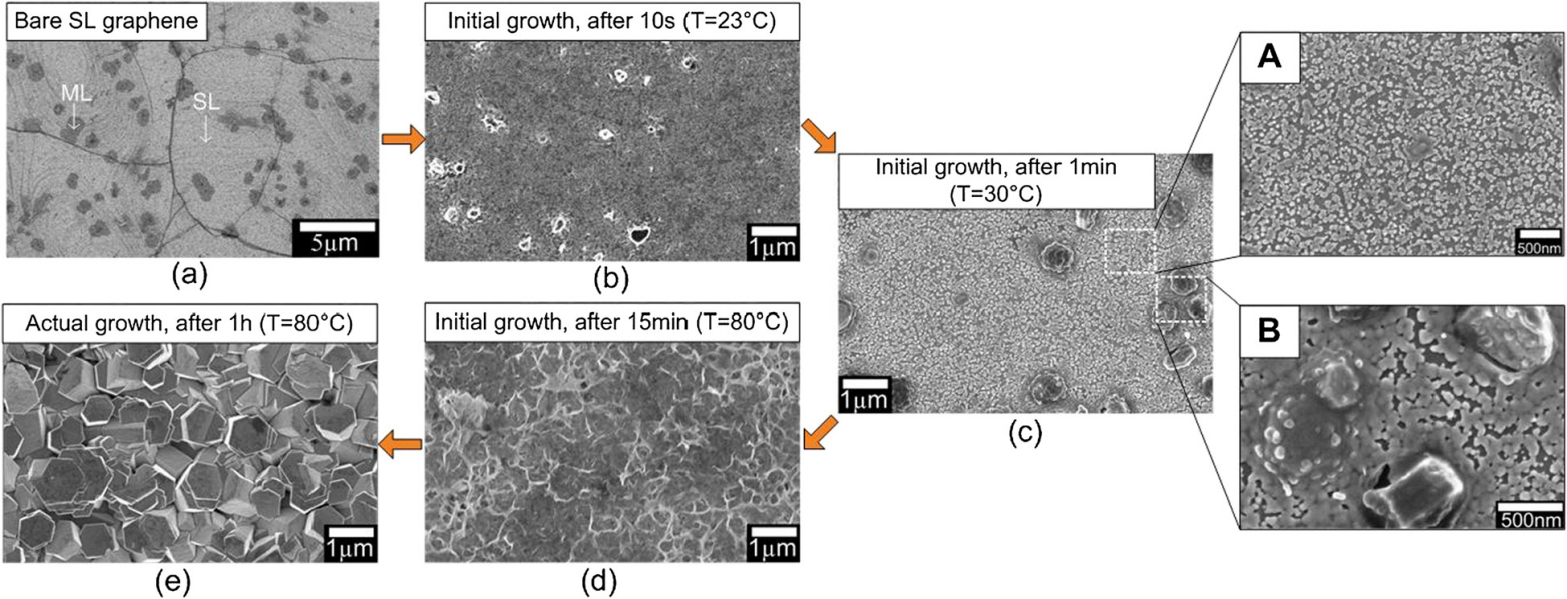
**FESEM images of bare SL graphene and ZnO structures grown on it at different growth times. (a)** Bare SL graphene. **(b, c, d)** ZnO structures grown on SL graphene after 10 s, 1 min, and 15 min of the initial growth, respectively. **(e)** ZnO structures grown on SL graphene after 1 h of the actual growth.

The results seem to prove that the nucleations are promoted at the stacking edges of ML graphene to form ZnO clusters and that the sizes of formed clusters increase with the increase of applied current density, resulting in the increase in sizes and diameters of rods and flower-shaped structures. To further prove this mechanism, we also perform a similar study using SL graphene. Figure 5a shows a bare SL graphene used in this work. It can be clearly seen that almost the entire surface shows the same bright color which corresponds to a single layer of graphene. However, there are some randomly distributed small dark spots which correspond to ML graphene. It is noted here that the substrate used consists of more than 95% coverage of SL graphene [44]. Similarly, the nucleation of ZnO starts to take place after 10 s of initial growth as shown in Figure 5b. However, the density of ZnO clusters was significantly small as compared to the ML graphene shown in Figure 4b. When the growth time is increased to 1 min, small ZnO spots with higher density were observed at the area of SL graphene as indicated by location A in Figure 5c. Moreover, it shows larger and thicker ZnO clusters at ML graphene as indicated by location B in Figure 5c. This observation seems to prove that the nucleation of ZnO is promoted at the edges of ML graphene. Again, as shown in Figure 4c, a very significant difference in the morphology can be clearly seen where the entire surface is fully covered with high-density ZnO structures with different thicknesses as compared to the morphology shown in Figure 5c. When the growth time is further increased to 15 min, a rough surface was observed but no rod or nanoflower-like structure was observed. Such observation was already discussed in our previous report [30]. In our previous report on the growth of ZnO nanostructures on SL graphene, the same procedures and experimental conditions were applied. In this case, we do not observe the growth of such flower-shaped structures on SL graphene [30]. As described in [30], the growth of vertically aligned/non-aligned rods as shown in Figure 5e observed after 1 h of the actual growth is due to the effects of surface roughness, high temperature of 80°C, and effective decomposition of HMTA.

In summary, the growth processes involve two main stages which are the formation of seed structure for nucleation sites of rods and flower-shaped structures below the ST point and the effective growth of non-aligned/aligned rods and flower-shaped structures after the ST point. These structures start to grow according to the shape of initial seed structures. Again, as proved by the FESEM images, the vertically aligned/non-aligned rods and flower-shaped structures are not growing directly on the graphene, but they are growing on the nucleation sites formed during the preheated process, i.e., below the ST point.

## Conclusions

In conclusion, seedless growth of highly dense vertically aligned/non-aligned ZnO rods and flower-shaped structures on ML graphene by electrochemical deposition was obtained. The applied current in the electrochemical system plays an important role in inducing the growth of ZnO structures on ML graphene as well as in controlling the shape, diameter, and density of structures. ML graphene seems to generate the formation of flower-shaped structures due to the multistacking structures. Such ZnO/graphene hybrid structures seem to provide several potential applications in sensing devices, etc.

## Competing interests

The authors declare that they have no competing interests.

## Authors' contributions

NSAA designed and performed the experiments; participated in the characterization and data analysis of FESEM, EDX, XRD, and PL; and prepared the manuscript. NIR participated in the data analysis and preparation of the manuscript. MRM participated in the PL characterization. KY and TN participated in the XRD characterization and revision of the manuscript. AMH participated in the monitoring of the experimental work, data analysis, discussion, and revision of the manuscript. All authors read and approved the final manuscript.
